# Supplementary material for: Contrast-Enhanced Spectral Mammography in the Evaluation of Breast Microcalcifications: Controversies and Diagnostic Management
Source: Healthcare (Basel). 2023 Feb 9;11(4):511. doi: 10.3390/healthcare11040511 (PMC9956946; doi:10.3390/healthcare11040511)
Supplement: Supplementary file 1 [file healthcare-11-00511-s001.zip › healthcare-2139461-supplementary.pdf]

Supplementary Table S1: Specific histological subtypes of the study breast lesions

Type of lesion	Benign / Malignant	Histological result	Overall (N=377)
**Microcalcifications**	**Benign and B3 lesions**	Adenosis	4
		Atypical ductal hyperplasia (DIN1b)	5
		Atypical lobular hyperplasia (LIN1)	5
		Breast fibroadenoma	2
		Ductal hyperplasia without atypia	4
		Fibrocystic breast disease	42
		Flat epithelial atypia	1
		Flogosis	2
		Intermediate grade lobular intraepithelial neoplasia (lin2)	2
		Intraductal papilloma	1
		Mucocele like lesion	1
		Pseudoangiomatous stromal hyperplasia (pash)	1
		Radial scar	1
	**Malignant**	High grade ductal carcinoma in situ (DIN3)	6
		Intermediate grade ductal carcinoma in situ (DIN2)	9
		Invasive ductal carcinoma	11
		Invasive lobular carcinoma	1
		Low grade ductal carcinoma in situ (DIN1c)	3
**No microcalcifications**	**Benign and B3 lesions**	Adenosis	7
		Atypical ductal hyperplasia (din1b)	1
		Breast fibroadenoma	8
		Ductal hyperplasia without atypia	2
		Fibroadenoma	2
		Fibrocystic breast disease	14
		Fibrosis	5
		Flat epithelial atypia	2
		Flogosis	5
		Intraductal papilloma	5
		Lobular intraepithelial neoplasia (lin2)	3
		Papillomatosis	1
		Radial scar	2
	**Malignant**	Acinic cell carcinoma	2
		Cribriform carcinoma	2
		High grade ductal carcinoma in situ (din3)	3
		Intermediate grade ductal carcinoma in situ (din2)	9
		Invasive cribriform carcinoma	2
		Invasive ductal carcinoma	152
		Invasive lobular carcinoma	32
		Invasive mixed carcinoma	10
		Low grade ductal carcinoma in situ (din1c)	4
		Malignant phyllode	1
		Micropapillary carcinoma	1
		Mucinous carcinoma	1
